# Extramedullary hematopoiesis presented as cytopenia and massive paraspinal masses leading to cord compression in a patient with hereditary persistence of fetal hemoglobin

**DOI:** 10.1186/s40364-016-0071-6

**Published:** 2016-09-01

**Authors:** Tasleem Katchi, Krishna Kolandaivel, Pallavi Khattar, Taliya Farooq, Humayun Islam, Delong Liu

**Affiliations:** Division of Hematology and Oncology, New York Medical College and Westchester Medical Center, Valhalla, NY 10595 USA

## Abstract

**Background:**

Extramedullary hematopoeisis (EMH) can occur in various physiological and pathologic states. The spleen is the most common site of EMH.

**Case presentation:**

We report a case with hereditary persistence of fetal hemoglobin with extramedullary hematopoiesis presented as cord compression and cytopenia secondary to multi-paraspinal masses.

**Conclusion:**

Treatment can be a challenge. Relapse is a possibility.

## Background

Extramedullary hematopoiesis (EMH) can occur in various physiological and pathologic states. The liver, spleen and yolk sac are the primary sites of hematopoiesis during fetal life. Additionally, the liver and spleen are also the sites of production of immune cells in response to antigen stimulation. The above are examples of EMH occurring in physiologic settings and can also be referred to as active hematopoiesis [1]. Extramedullary hematopoiesis also occurs in pathologic processes such as myelofibrosis, where the bone marrow is replaced with fibrous tissue and can no longer carry out adequate hematopoiesis to meet the demands of the body. This is referred to as passive hematopoiesis [2].

Several sites of extramedullary hematopoiesis (EMH) have been reported in the literature. The common ones are spleen, liver, lymph nodes, and thymus. Rarely, EMH has been reported in the cardiac tissue, breasts, renal tissue, adrenal glands, pleura, retroperitoneal tissue, skin, prostate, broad ligaments, peripheral and cranial nerves, and the spinal canal [3–5]. Reports indicate that the dura mater may have hematopoietic capacity during fetal life which becomes re-activated in pathologic states [6]. Other theories include extrusion of hematopoietic tissue from the verterbral body or the proximal rib ends [7, 8]. Development of hematopoietic tissue in the intercostal veins with subsequent embolization has also been suggested [9].

The occurrence of spinal cord compression secondary to EMH is extremely rare and very few cases have been reported in the literature. The first case was reported by Close et al. about 60 years ago in a case of thalessemia minor [10]. The most common cause for spinal EMH is thalassemia. Other rare causes include pernicious anemia, myeloproliferative neoplasm such as polycythemia vera, sideroblastic anemia, pyruvate kinase deficiency, and myelofibrosis disorders [11–16]. Symptomatic spinal cord compression secondary to EMH showed a preference for localization in the middle and lower thoracic spine. Some authors related this to the narrow spinal diameter at this level [17–19]. Here we report a case of EMH presented as cytopenia and cord compression.

## Case presentation

The patient is a 51 year-old female who presented with progressively worsening bilateral lower extremity numbness and gait difficulty for 2 months. Her medical history was significant for stage I breast cancer which was treated with wide-excision lumpectomy followed by radiation therapy. No hormonal therapy nor chemotherapy was administered.

On physical examination, her vital signs were stable and there were no focal cardiac, respiratory or abdominal findings. There was no icterus or palpable lymphadenopathy. There was no palpable hepatosplenomegaly. Her neurological examination revealed intact motor strength and normal tone throughout. There were no cranial nerve or cerebellar deficits. There was diminished sensation to light touch and pin prick at T5-T6 level. Laboratory examination showed a haemoglobin (Hb) level of 10 g/dL, mean corpuscular volume of 78.1 fL, leucocyte count of 10,300/μL, and platelet count 125,000/μL. Liver and renal function tests were also normal. LDH was 272 U/L (range 125-220) at presentation.

The patient had relatively low reticulocyte count, normal iron studies, folate and vitamin B12 levels. Hb electrophoresis revealed Hb A2 (alpha 2 delta 2) 3.8 % (normal range 1.5-3.5) and elevated fetal hemoglobin (alpha 2 gamma 2) HbF 38 % (range 0.0-2.0). Urine and serum protein electrophoreses were unremarkable. Bone marrow karyotyping showed 46 XX. Alpha-globin gene analysis for alpha thalassemia was negative.

**Fig. 1:**
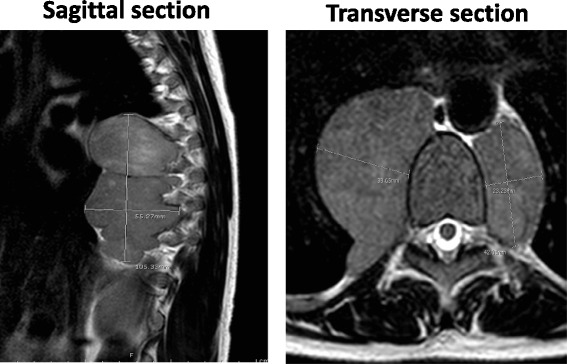
MRI study of the paraspinal masses. Representative images of the paraspinal masses were shown

MRI of the entire spine revealed abnormal signals of the cervical, thoracic and lumbar vertebrae. A heterogeneous predominantly T2 hyperintense and T1 isointense enhancing lesion (9.7× 1.6× 1.0 cm) extending from the T4 level to the T11 level with near complete effacement of the spinal canal and increased T2 signal within the spinal cord compatible with T5-T6 spinal cord compression. In addition, there were large bulky bilateral enhancing paraspinal lesions from T6 extending to T12 (10.5 × 5.5 × 4.0 cm on the right and 5.0 × 4.2 × 2.2 cm on the left) (Fig. 1). There was a soft tissue presacral mass ajacent to S5 measuring approximately 2.0 × 2.0 × 1.1 cm.

CT scan of the chest/abdomen/pelvis revealed multiple masses in the right hepatic lobe and a large left hepatic lobe mass with nodular peripheral enhancement suspicious for hemangioma, mild splenomegaly, cystic pancreatic head mass, upper retroperitoneal lymphadenopathy, large uterine mass and large anterior abdominal wall mass. The differential diagnosis at this point was metastatic disease with unknown primary, likely breast vs. primary gynecologic malignancy.

**Fig. 2:**
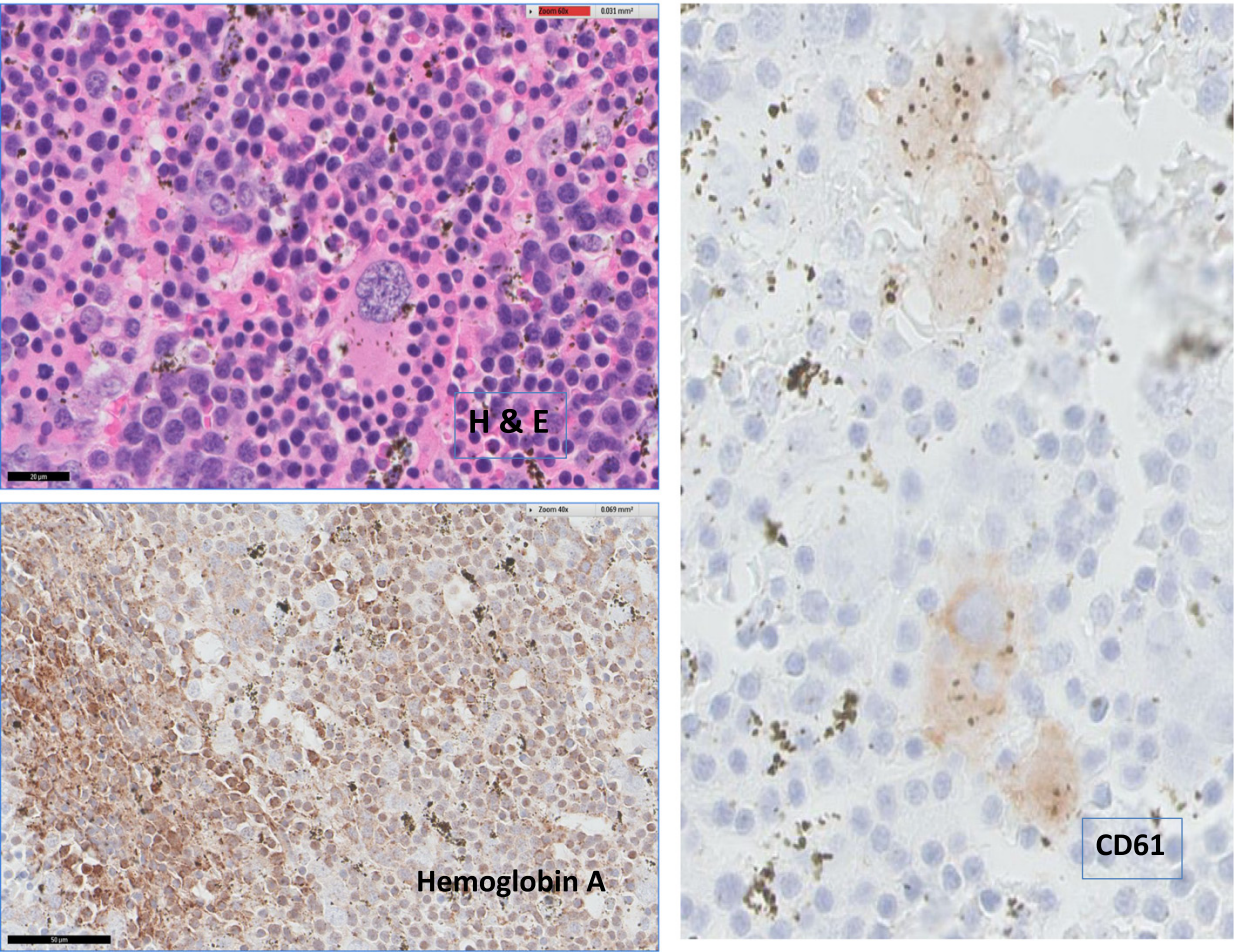
Pathology of the thoracic paraspinal mass. The thoracic paraspinal mass was resected. H & E stain reviewed dense hematopoietic tissues. Immunostaining was positive for hemoglobin A (red cell) and CD61 (megakaryocytes)

The patient underwent a bone marrow biopsy which revealed hypercellular bone marrow with erythroid hyperplasia. Flow cytometry study was negative for leukemia and lymphoma. Bone marrow core biopsy was negative for metastatic disease. For relief of neurological symptoms and final tissue diagnosis, patient underwent T4-T9 total laminectomy and removal of the thoracic epidural tumor. Intra-operative frozen section revealed suspicious lymphoproliferative process. Final pathology of the resected mass revealed dense hematopoietic tissue with erythroid hyperplasia, scattered megaloblastic forms and megakaryocytes, entrapped bony spicules, intervening fibrous septa and adjacent scant adipose tissue (Fig. 2). Immunohistochemistry was negative for cytokeratin AE1/AE3, CD20, CD30, CD1a, and ALK-1. The tissues were positive for hemoglobin A and CD61. Therefore, there was no evidence of leukemia, lymphoma, or other malignancies. These morphologic findings were consistent with extramedullary hematopoiesis.

The final diagnosis in this patient was EMH along the paraspinal areas leading to cytopenia and cord compression.

MRI of the liver was obtained in view of the hepatic mass seen on CT scan of the abdomen and elevated transaminases that the patient developed during the hospital course. MRI findings was compatible with liver hemangioma and the transaminitis was attributed to liver hemangioma versus EMH. Due to the extensive nature of the masses along the bilateral paraspinal areas and cord compression, the patient underwent radiation therapy. The patient had steady improvement and regained muscle strength after rehabilitation.

## Discussion

This case represents an unusual extensive EMH causing cord compression and cytopenia. The patient appeared to have hereditary persistence of fetal hemoglobin with HbF 38 % at diagnosis. There was no evidence of sickle cell anemia nor thalathemia. It is not entirely clear what pathological process in the bone marrow led to the EMH. It is equally ambiguous as to what initiated and supported the extensive bilateral paraspinal EMH. Due to the extensive EMH with multiple bulky masses, radiation was chosen to eliminate the EMH.

Several mouse models were established to study the possible molecular events leading to EMH [20–22]. There was no molecular mutation that was found and no evidence of myelofibrosis in the bone marrow in this case. This case and a few of previous reports on paraspinal masses with EMH may suggest that there are supporting tissues/stromal cells that allow hematopoietic stem cell niches to survive and propagate in the paraspinal spaces. Adipose tissues and splenic stromal cells have been reported to support EMH [23, 24]. Since the pathogenesis and the cause of the extensive EMH were uncertain, it remains unknown whether EMH in this subject will recur.

## Conclusion

Hereditary persistence of fetal hemoglobin was associated with EMH in this case who presented as cytopenia and cord compression secondary to multi-paraspinal masses.

## Abbreviation

EMH: Extramedullary hematopoiesis
